# New Considerations Around the Singular Water Temperature Explaining the Anomalous Behavior of Liquid Phase

**DOI:** 10.3390/ijms27031606

**Published:** 2026-02-06

**Authors:** Domenico Mallamace, Giovanni Romanelli, Roberto Senesi, Francesco Mallamace

**Affiliations:** 1 Department of Chemical, Biological, Pharmaceutical and Environmental Sciences (ChiBioFarAm), University of Messina, 98166 Messina, Italy; mallamaced@unime.it; 2 Physics Department, Università degli Studi di Roma Tor Vergata, Via della Ricerca Scientifica 1, 00133 Rome, Italy; giovanni.romanelli@uniroma2.it (G.R.); roberto.senesi@uniroma2.it (R.S.); 3 ISIS Neutron and Muon Source, Rutherford Appleton Laboratory, Chilton, Didcot OX11 0QX, UK; 4 Istituto di Struttura della Materia (ISM), Consiglio Nazionale delle Ricerche, Via del Fosso del Cavaliere, 100, 00133 Rome, Italy; 5 Dipartimento di Scienze Matematiche e Informatiche, Scienze Fisiche e Scienze della Terra (MIFT), Università di Messina, 98166 Messina, Italy

**Keywords:** water, mestable supercooled regime, polidispersity, thermodynamical functions

## Abstract

The water thermodynamics is characterized by polydispersity, which determines its structural and dynamic properties. This is due to the specifics of its characteristic bond: the hydrogen bond (HB). The isobars of the two fundamental thermodynamic functions, the isothermal compressibility ($K_{T}\left(P.T\right)$) and the isobaric expansivity ($\alpha_{P}\left(P,T\right)$), show the presence of a temperature $T^{*}≃315\pm 5$ K where both have a singular behavior. In this work, by carefully considering the thermal properties of the isobars of density ρ, specific heat $C_{P}$ and the self-diffusion $D_{S}$, we suggest the universality characteristics of this temperature. In addition, by analyzing the average intermolecular distance $d_{OO}$, in the same area of the P-T phase diagram, we demonstrate that such realities are due in the supercooled liquid state to the ratio between its two characteristic phases: the low-density liquid (LDL due to HB) and the HDL (which entirely characterizes the remaining parts of the phase diagram).

## 1. Introduction

As is well known, water shows chemical and physical diversity when compared to other liquids, so it is not yet fully rationalized. Although it has a simple molecular structure, water has different chemical–physical characteristics than other substances [1]. For these intriguing reasons, this liquid has always been the focus of various careful studies. Furthermore, if the corresponding studies are of interest, in science and engineering, water can be explained only by the use of physico-chemical models and experiments, in particular that of the thermodynamic and of the modern statistical physics [2]. Starting from the well-known density maximum, observed under the ambient pressure (0.1 MPa) at 4 °C, unusual properties of the liquid phase of water include the minima in the *T* dependences of the isothermal compressibility ($K_{T}$) and isobaric specific heat ($C_{P}$) located at about 40 °C. Similar behaviors are also observable in the water thermodynamic functions like the self-diffusion coefficient ($D_{S}$) that near the ambient temperature increases its growth in response to an applied pressure (unlike the corresponding density (ρ)).

Like some other liquids, water can be easily supercooled below its melting temperature ($T_{M}=273.17$ K), where it shows unusual dynamics with characteristic divergent behaviors for which is as a sort of prototype of glass-forming systems. The limit of this supercooling region is the homogeneous nucleation temperature ($T_{H}≃232$ K for P=0.1 MPa) at which the nucleation rate suddenly becomes very large. However, such a limit, being of a kinetic nature, is not absolute and can be bypassed if the observation time is shorter than the nucleation time. At lower temperatures, a highly viscous liquid phase (also metastable) can be obtained at about 150 K ($T_{X}$) by heating water to the glassy state; liquid that crystallizes as cubic ice when *T* is further reduced. In such a situation, extremely high viscosity causes the nucleation rate to slow down, allowing much longer observation times. So $T_{H}$ and $T_{X}$ are then the limits of a region inaccessible to experiments on the liquid in its metastable phase. These limits can be overcome by using confined water, water in man-sized pores, or emulsified water. By choosing the confinement pore size smaller than its homogeneous nucleation process, water does not crystallize and can be further cooled below $T_{H}$.

Thus, by using confining sizes of the order of tens of micrometers, it was possible to extend the experimental studies of the bulk water thermodynamics in a large region of its *P*-*T* phase diagram. The pioneer of this idea was Angell with collaborators, who detailed the behaviors of $K_{T}$ and $C_{P}$ in the region $T<T_{M}$. A goal of significant interest in the obtained results is that by showing specific power laws they proposed the presence of a critical behavior [3].

Water in the solid crystalline phase posses another relevant characteristic: the polymorphism, characterized by many ice structures ranging from the ice Ic to ice XII (all of different density) [4].

Mischima studies on amorphous water, clearly highlighting also the glass polymorphism, have provided new information on liquid properties [5,6]. IIn fact, a real polyamorphism was discovered in the system, with the transition governed by the *T*, between the two amorphous forms of different densities, i.e., from high (HDA) to low (LDA). The latter, with ρ≃0.94 gcm^−3^ similar to that of the ice I_*h*_, was discovered in 1935 [7], whereas HDA can be achieved, at 77 K, up to 2 GPa, by compressing ice I_*h*_, and it has a density of ∼1.17 gcm^−3^ [8]. In addition, if heated, at 0.1 MPa, it passes into the low-density form at ∼120 K. Such transition is reversible (HDA ⟷ LDA) as is the one between LDA and HDA by pressure-cycling at about 135 K and 2 GPa) [9]. As said, the LDA, when heated, transforms into highly viscous liquid.

An additional higher-density glass form has recently been discovered [10]: the VHDA or very-high-density amorphous [11]. The extension of this “polyamorphism”, with these singular amorphous forms, in the liquid phase has allowed us to clarify the system thermodynamics. Like in glass, the liquid polymorphism is characterized by two phases different in densities: the high- and low-density liquids, HDL and LDL [12]. While the latter is more relevant from a physical point of view, for its “open” structure originated by a clustering with a tetrahedral symmetry due to the non-covalent attractive hydrogen bonding (HB) interaction, the former is instead made up of a rather homogeneous distribution of trimers, dimers, or single water molecules. These two liquids coexist and depending on the thermodynamic variables can transform into each other through a first-order transition: the liquid–liquid transition hypothesis (LLT) [13,14,15,16,17]. All these water experiments are proven realities: polyamorphism, the continuity between its glass and liquid accompanied by a discontinuity between the two liquid forms, HDL and LDL (the liquid–liquid transition, LLT) are at the bases of the liquid–liquid critical point (LLCP) hypothesis (an additional critical point in addition to the vapor–liquid one) [18]. Three interactions, one repulsive and two attractive, govern the water properties: the first is the purely electrical Coulomb force between electron lone pairs of adjacent oxygen atoms; the attractive ones are the covalent bond CB (*O*-*H*) and the hydrogen bond HB (*O*-*H*).

The single molecule (H_2_O) originates from two CBs sharing the electron pairs with a binding energy BE ≃4 eV and filling up other two orbitals by means of its non-bonding lone pairs originating the intermolecular non-covalent, *O*:*H*, van der Waals bond (0.1 eV). Three distances are representative of its structure (two intermolecular $d_{OO}$ and $d_{O:H}$ and the intramolecular *d*_*O-H*_) and two angles (the internal *H*-*O*-*H* (θ) and intermolecular *H*:*O*-*H* (φ), depending on both pressure and temperature. In general, the Bernal and Fowler ice rule holds and the oxygen atom always tends to find four neighbors to form a stable tetrahedron (θ<104.5°,φ>109.5°) whereas, on the contrary, in the liquid phase, the repulsion between electron pairs on oxygen counteract the steady tetrahedron formation. In water, such a basic building block contains two equivalent molecules and four identical *O*:*H*-*O* bonds (with different orientations). Despite the fluctuations in φ and $d_{O:H}$, its average structural dimensions are independent of the thermodynamic transitions that govern water, from solid to liquid phase or to glass or vapor one. Summarizing, while HB dominates the liquid in both the stable and metastable regimes (supercooled), the repulsion mainly influences the water properties from above the boiling temperature ($T_{b}$) up to the sub-critical and critical region. Finally, the vapor–liquid critical point CP coordinates are $T_{C}=647.1$ K, $P_{C}=22.064$ MPa, whereas the LLCP is estimated by Molecular Dynamics simulations to be at 200 K and 200.064 MPa [19].

The observed behaviors of some thermodynamic functions of water, such as their divergences, and the decrease in entropy accompanied by polymorphism (especially that of the liquid phase) are at the origin of its complexity and related anomalies. It should be noted, however, that through these behaviors, the presence of local tetrahedral order has been experimentally demonstrated. Unfortunately, in spite of the proper theoretical suggestions and the very large number of accurate computational studies accompanied by proper suggestions [19], the suggested liquid criticality in the metastable (supercooled) phase is far from being proven [20]. Today, such a result is an intriguing chimera for experimental physics.

Despite this, we still have some certainties observed in the liquid water when it is cooled, such as the growth in size and stability of the tetrabonded clusters. Conditions for which the HB lifetime strongly increases by many orders of magnitude from the some picoseconds typical of the stable liquid region ($T>T_{M}$) [21]. In contrast to the permanent ice tetrahedral network, the liquid tetrahedrality is local and transient. An increase in pressure opposes these ordering processes.

As said, bulk liquid water cannot exist in the region between the homogeneous nucleation temperature ($T_{h}$) and that in which the ultra-viscous liquid crystallizes ($T_{x}$): the so-called “No Man’s Land” water. But such a constraint can be overcome by using confined water so that water can be maintained in the liquid state inside the range $T_{h}$–$T_{x}$ and thus explored [22].

Other ways to work inside this region is to use water in solutions, that of hydration of macromolecules and in micellar systems [23], nano droplets [24] inside and outside ice, or by melting a multi-molecular thickness of an ice surface [25]. Just these approaches have led to the discovery of new and important properties that characterize it, like the density minimum [26,27], predicted by Percy W. Bridgman [28] and confirmed by proper computational studies [29,30,31]. Regarding these reported MD data, we stress that they come from different models (TIP4P or E3B3) largely used for the water system [19].

Other important findings concern the dynamics: the crossover from a fragile to a strong glass-forming material, predicted at ambient pressure [32] and observed at $T_{L}≃225$ K [33]; in the P-T phase diagram, this is also the locus of the Stokes–Einstein relation violation (due essentially to the decoupling between translational and rotational modes) and of the Widom line that, strongly linked to the LLCP hypothesis, identifies the maximum in the δV and δS fluctuations where thermodynamic response functions reach their extremes (minimum with negative values in the $\alpha_{P}$ and maxima in $C_{P}\;$ and $\kappa_{T}$).

**Figure 1:**
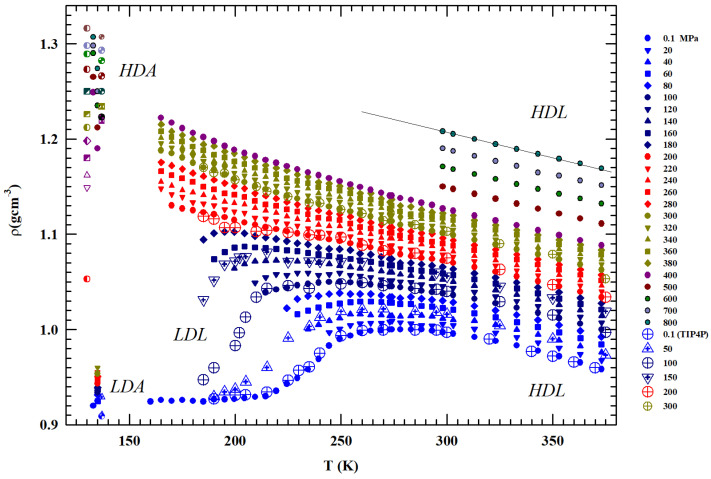
The measured liquid water densities, ρ (gcm^−3^), in temperature range 100<T<400 K for several pressures from 0.1 to 800 MPa. The values corresponding to the low- and high-density (LDA and HDA) amorphous phases measured at 80, 130 and 138 K are also included together with those obtained by means of molecular dynamic simulation (TIP4P). The figure also includes values measures for P=0.1 MPa under confinement just to explore liquid water in the deep supecooled regime. The low-density liquid and high-density liquid regions are also indicated.

Here, by using the liquid and amorphous water density literature data, reported in Figure 1 in a wide P-T range, we focus on the HB role and effects (structural and dynamical). Previous studies (see, e.g., [20]) have detailed that the LDL is present (together with HDL) only in a limited phase diagram area, where it is responsible of the LLT and the corresponding anomalies, whereas in the remaining parts, essentially only the HDL exists. Also from the reported data, the existence of a limiting pressure is evident above which the liquid is present only as HDL and freezes only into HDA, whereas at lower pressures, where HDL and LDL phases coexist, the liquid evolves only toward the LDA. Using these ρ(P,T) data, we evaluate the *T*—dependences of two fundamental thermodynamic functions, the isothermal compressibility ($K_{T}\left(P.T\right)$) and the isobaric expansivity ($\alpha_{P}(P,T$)), at different isobars discovering the presence of a temperature $T^{*}≃315\pm 5$ K in which both show a singular behavior: all the compressibility isobars have a minimum value and the corresponding $\alpha_{P}$ functions cross each other [34]. The “singular and universal expansivity point” is $\alpha_{P}\left(T^{*}\right)≃0.44\times 10^{-3}$ K^−1^.

Furthermore, according to the results of NMR studies, it has been highlighted that this temperature defines the boundary point between two regions of different energy configurations and therefore different dynamics. Specifically, for $T>T^{*}$ the water properties are governed by two defined energy levels (the Arrhenius behavior) while in opposite thermal conditions ($T<T^{*}$) the situation changes completely, becoming that which characterizes supercooled glass-forming liquid systems. Below $T^{*}$, cooling gives rise in water to specific correlations in the time and length scale, resulting in a dynamical clustering, i.e., the onset of the HB tetrahedral network and its effects [35]. Starting from these results, in this work, we aim to demonstrate the universality of this temperature in characterizing the physics of water in its structural and dynamic properties.

## 2. Results and Discussion

### 2.1. The Density

Figure 1 illustrates the bulk water densities (ρ(P,T)) for 1 < P (MPa) <800 and 100< T (K) <380 K [36,37,38,39,40,41,42]; the values measured in the LDA and HDA glass are also reported, as well as the data of a MD simulation [29,30] together with the data measured at ambient pressure (P=0.1 MPa) for water under MCM nanotubes confinement and emulsifieds (droplet size 1–10 μm) [42].

Being *P*-dependent, the density maximum disappears for P>180 MPa. Such pressure also identifies the change in the curvature of the density isobars (${\left(\partial \rho /\partial T\right)}_{P}$) from negative to positive. The densities measured at 130 and 150 K in the HDA and LDA are also shown. Whereas the HDA data measured at very high pressures are of the order of 1.25 gcm^−3^ (or even higher), the value of the corresponding LDA for 0.1 MPa and 135 K is ∼0.94 gcm^−3^. The latter value agrees with the values measured in confined water (nanotubes) inside the range $T_{h}$–$T_{x}$, where a minimum is present at T∼200 K [26].

From the reported data, it can be observed that (i) the ρ(P,T) grows with pressure and such increase is larger in the metastable supercooled phase; (ii) the data spread at a fixed temperature is lager just in the region of the two amorphous phases (e.g., at 130 K is $\Delta \rho^{130}\left(T\right)≃0.42$) rather than in the heated liquid (at 400 K is $\Delta \rho^{400}\left(T\right)≃$ 0.22; (iii) as said, for *P* ≥ 200 MPa, the curvature changes from concave to convex with an evolution similar to that of a simple fluid; (iv) the pressure of 200 MPa defines two different behaviors: all the isobars below such pressure density evolves toward the LDA values, while all those above it proceed to the HDA; (v) whereas the LDA densities are almost P independent, those of the HDA phase show a marked dependence on this variable.

### 2.2. The Thermodynamic Density Derivatives (The Isothermal Compressibility and the Isobaric Thermal Expansivity)

**Figure 2:**
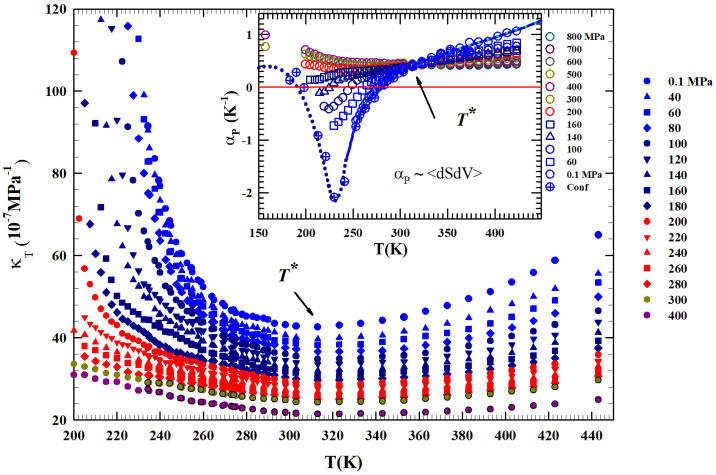
The liquid water isothermal compressibility $K_{T}\left(T,P\right)$ obtained from the density data in the liquid phase illustrated in Figure 1 for *P* in the interval 0.1–400 MPa for the range 200<T<450 K. The corresponding values of expansivity $\alpha_{P}$ are reported in the inset. It is clearly observable that all the compressibility isobars show a minimum just at $T^{*}$ while those of $a_{P}$ all cross in a single point located exactly at the same temperature ($T^{*}≃315\pm 5$ K).

Figure 2 shows, from 200 to 450 K, the different isobars (0.1 to 400 MPa) of the isothermal compressibility, K_*T*_(T,P), obtained as $K_{T}=-V^{-1}{\left(\partial V/\partial lP\right)}_{T}={\left(\partial \ln\rho /\partial \ln P\right)}_{T}$, a function that also represents the volume fluctuations δV as $K_{T}={\langle \delta V^{2}\rangle }_{P,T}/k_{B}TV$. It is clearly observable that all the obtained compressibility isobars show a minimum just at $T^{*}$. In addition, an increase in the corresponding values for $T<T^{*}$ can be observed for all isobars compared to the second region at higher temperatures. This growth is increasingly marked in the metastability (supercooled) regions, especially for the isobars from 0.1 to 200 MPa, while at higher pressures (P>200 MPa) the resultig compressibility values are much lower and almost symmetric in temperature around the minimum in $T^{*}$.

The same Figure proposes in the inset the isobaric expansivity $\alpha_{P}=-{\left(\partial \ln\rho /\partial T\right)}_{P}=-V^{-1}{\left(\partial S/\partial P\right)}_{T}$, linked to the entropy–volume cross-correlations 〈δSδV〉 to be $\alpha_{P}=\langle \delta S\delta V\rangle /k_{B}TV$. This function is proposed for 150< T(K) <450 K and pressures from 0.1 to 800 MPa.

**Figure 3:**
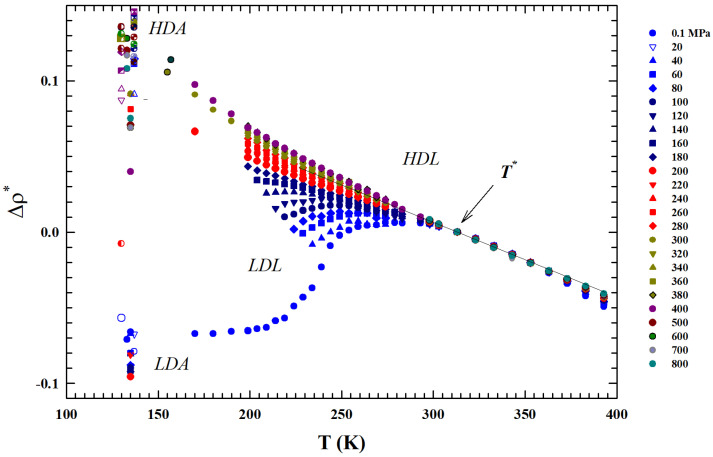
The figure shows all the experimental density isobars reported in Figure 1 normalized by subtraction to the corresponding $T^{*}$ values ($\Delta \rho^{*}$). The obtained isobars are shown in the same range of Figure 1: 100< T (K) <400 K. The reported data also highlights that in the inteval $T^{*}<T<T_{b}$ the thermal effect on for all the density isobars is the same.

Regarding $\alpha_{P}$, at ambient *P* in simple liquids, the δS and δV fluctuations become smaller on decreasing *T* and are positively correlated. In water, the situation is opposite: they are not only more pronounced, but in the region of metastability (T<277 K) they are anti-correlated [43], a behavior occurring due to the evolution of the water local order in the supercooled region. Furthermore, the thermal evolution of both these functions, compressibility and $\alpha_{P}$, shows that $T^{*}$ is a border between two different behaviors. All the $\alpha_{P}\left(T\right)$ isobars cross, within the error, just at $T^{*}$. Even in this case we see that above this temperature the thermodynamic behavior of water is exactly the same as a normal fluid, but the situation changes on cooling, where anomalous behaviors are evident. More precisely, with P at 200, 300, and 400 MPa, a continuous evolution occurs at all temperatures. But at the lowest pressures (P<200 MPa) for $T<T^{*}$, the $\alpha_{P}\left(T\right)$ evolution becomes more complex, i.e., it decreases as *T* decreases and, as proposed by the P<160 MPa isobar, the entropy–volume anticorrelations determine an inflection point and its minimum. This minimum decreases in value as *P* increases, a behavior consistent with the density minimum observed in confined water. Note that in the case of confined water (MCM-41), such minimum temperature is coincident with that of the fragile-to-strong dynamic crossover and the Widom line, which at ambient pressure is $T_{W}\left(P\right)≃225$ K. We have to stress that the water singular temperature $T^{*}$ has a precise thermodynamical consistence lying in the relationship connecting two of the studied response functions:

(1) $${\left(\frac{\partial \alpha_{P}}{\partial P}\right)}_{T}=-{\left(\frac{\partial K_{T}}{\partial T}\right)}_{P},$$

a thermodynamic rule that confirms the $T^{*}$ role for the liquid water [34]. Just to show the uniqueness of this temperature and the marked difference in the isobar behavior above and below it, also by considering the suggestions of these two thermodynamic response functions, we normalized the data of the isobars reported in Figure 1 by subtracting for each one the corresponding density value $\rho_{P}\left(T^{*}\right)$. The obtained result ireported in Figure 3 shows another special feature: from $T^{*}$ up to $T_{b}$, the thermal effect on for all the density isobars is the same (and linear).

### 2.3. The Constant Pressure-Specific Heat

**Figure 4:**
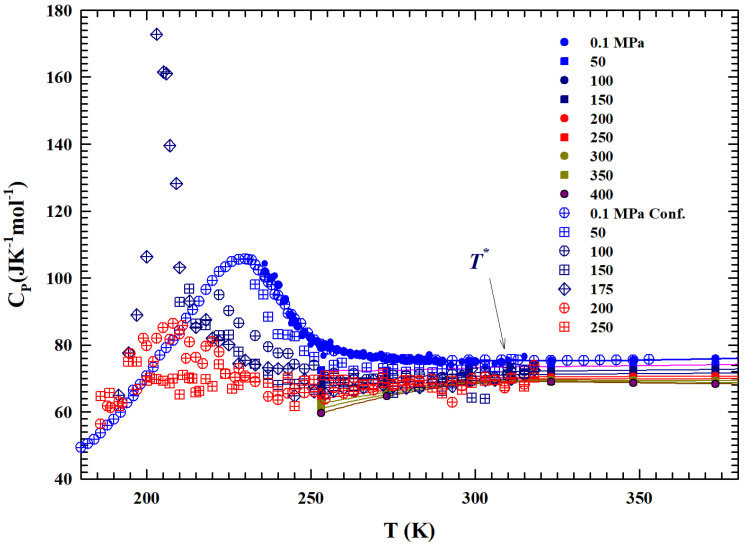
The figure illustrates, in the temperature range of 180–380 K, the isobars of the specific heat $C_{P}\left(T,P\right)$ of the bulk liquid water from 0.1 to 400 MPa [44,45,46,47]. Open symbols represent data meaured in confined water MCM 10 μm. Almost all of the data in the supercooled metastable region, characterized by sharp maxima, are obtained from confined water.

Figure 4 shows, in the range of 180<T<380 K, two sets of the constant pressure-specific heat isobars, $C_{P}\left(P,T\right)$.

The first one illustrates the experimental measurements obtained with a differential calorimeter [44,45] (at 0.1 MPa) and sound velocity [46]; the second instead presents data obtained from NMR time relaxation [47] data through the use of the Adam–Gibbs relation [48]:

(2) $$D_{S}=D_{S0}\exp\left(\frac{A}{TS_{Conf}}\right)$$

with the *A* constant. Data fitting in the range of bulk water gives $D_{S0}=1.07$ × $10^{-7}$ $\;m^{2}$ $\;s^{-1}$, whereas *A* is estimated to be −31.75 kJ mol^−1^; these values are found to be in agreement with those of ref. [49]. The corresponding values of $TS_{Conf}$ and $S_{Conf}$ are thus obtained. Hence, $C_{P,conf}$ is obtained by means of the derivative $C_{P,conf}=T{\left(\partial S_{Conf}/\partial T\right)}_{P}$. From the temperature behavior of all the $C_{P}$ isobars, a slope change can be observed at about $T^{*}$. The data behavior is characterized by a maximum (e.g., for 0.1 MPa at about 225 K) in the $C_{P,conf}$ measured in confined water. A coincidence of the values can also be observed, within the experimental error, between those evaluated by the self-diffusion coefficients and those experimentally measured in liquid.

### 2.4. The Self-Diffusion Coefficient

**Figure 5:**
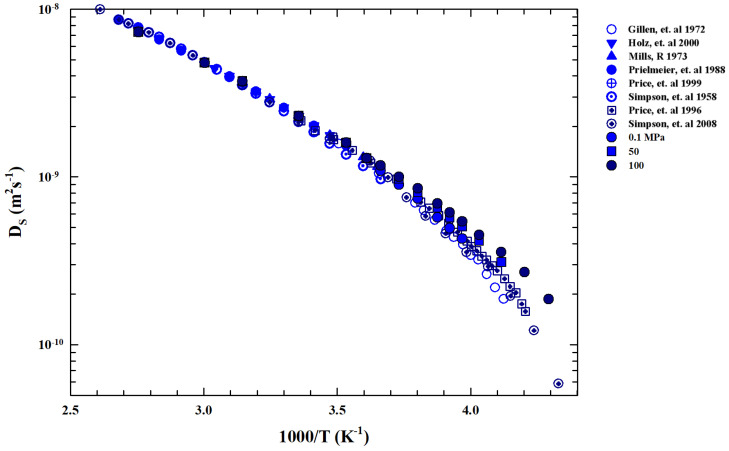
Arrhenius plot of the liquid water self-diffusion data $D_{S}$ ($m^{2}$$s^{-1}$) coming from different experiments [21,50,51,52,53,54,55,56] in the range of 196<T<383 K in the pressure region (0.1< P(MPa) <100).

We now examine the self-diffusion coefficient $D_{S}\left(P,T\right)$ data in order to gain further information about $T^{*}$. Figure 5 shows an Arrhenius plot of the bulk water $D_{S}$ measured in different experiments. Function of pressure (0.1< P(MPa)<100) is in the range 373< T(K) <−235.

Data are collected from Nuclear Magnetic Resonance (NMR) experiments [21,50,51,52,53,54,55,56]. For $T>T^{*}$, the reported data evolve according to the Stokes–Einstein relation, whereas below we show a super-Arrhenius behavior according to the universal one corresponding to the materials liquid state [57]. In particular, for water, $T^{*}$ represents the crossover temperature marking the system dynamic crossover from simple two energetic states (Arrhenius or strong glass forming liquids [58]) to the super-Arrhenius (or fragile liquids) characterized by an energetic multi-state distribution, which in turn is characterized by dynamic heterogeneities (with a multi-relaxation in the time evolution of the density–density correlation functions) typical of the supercooled regime and directly linked with $T^{*}$ [59,60].

### 2.5. The Rotational Relaxation Time

**Figure 6:**
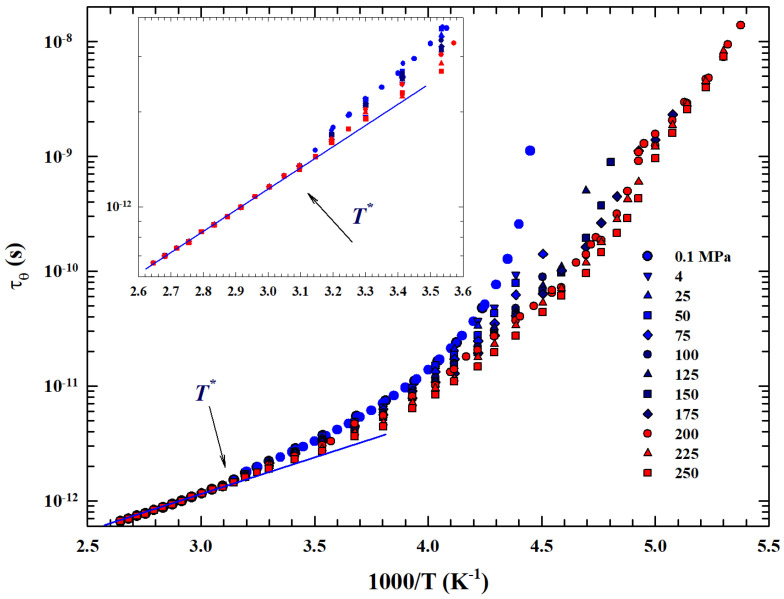
Arrhenius plot of the *T*-dependence of water rotational relaxation time $\tau_{\theta }$ measured by means of the NMR technique in the large temperature range 186–370 K. Data illustrated for several isobars from 0.1 to 250 MPa.

The general validity of this universal-like crossover is confirmed by the rotational relaxation time data, $\tau_{\theta }\left(P,T\right)$, at different temperatures and pressures reported in an Arrhenius plot in Figure 6. Temperature data are considered from the boiling point down to 185 K for many pressures, as indicated in the legend from 0.1 to 250 MPa.

In particular, data coming from an NMR experiment are illustrated, measured by means of the system longitudinal relaxation times $T_{1}$ in emulsioned water contained in a high-pressure glass capillary [47]. Hence, the $\tau_{\theta }$ values were evaluated by taking into account only the inter- and intramolecular dipolar contributions observed in the proton relaxation as ${\left(1/T_{1}\right)}_{obs}={\left(1/T_{1}\right)}_{inter}+{\left(1/T_{1}\right)}_{intra}$ according to proper procedure [61]. As can be easily observed also for $\tau_{\theta }$, in accordance with what has been said, $T^{*}$ represents a significant crossover between two distinct behaviors. In addition to the strong–fragile glass-forming transition, a clear dependence on pressure is evident within the experimental errors before and after this temperature: (i) for higher temperatures the obtained rotational relaxation time values are *P*-independent, while (ii) below $T^{*}$ the dependence on P is increasingly significant, increasing both with decreasing *T* and increasing *P*.

Finally, we considered that $T^{*}$ marks a transition from a *T* region, where the water dynamics is governed by only two energy levels with a single relaxation process (the Arrhenius configuration), to another typical glass-forming liquid in which temperature decrease originates large intermolecular correlations. In the time and length scale, this lead to dynamic clustering (the HB tetrahedral network) with a large distribution of possible energies and thus multirelaxations (a super-Arrhenius system).

Hence, like in complex liquids, this complex energetic situation determines a behavior for all the transport parameters of water (relaxation times, viscosity, and self-diffusion) characterized by this specific dynamic crossover, a situation unanimously proposed by the liquid state theories of crossover temperature that marks the transition from normal to supercooled liquid [62,63,64].

Physical reality is observable in the dynamics of such systems, that is, both in their time and relaxation processes; in the case of water, these thermodynamic realities are correctly associated to HB interactions. These interactions give rise to specific transient structures that become increasingly stable as the material cools within the metastability region. In the case of liquid water, all of this originates at $T^{*}$ via the HB clustering process. This temperature is therefore the magic point where the water system becomes the well-known intriguing and complex material. In our opinion, only by considering the dynamical quantities reported in this work in a unified manner can the importance of this temperature be fully appreciated. Hence, as said, all the reported data measured in the large *P*-*T* phase diagram considered and the consistency proposed by the response functions $K_{T}$ and $\alpha_{P}$ indicate that $T^{*}$ plays a primary role in the water physics and it is the locus where its anomalies originate.

For these reasons, we conclude our analysis by directly looking at the liquid water local HB structure effect on the oxygen–oxygen distance ($d_{OO}$), evaluated from the density data reported in Figure 1, for temperatures below 373 K up to the deep supercooling regions.

### 2.6. The Intermolecular Oxygen–Oxygen Distance

The water molecules possess high asymmetry with coordination numbers ranging from two to four or even greater [65]. The water’s liquid structure, as said, can be interpreted in terms of either tetrahedrally coordinated structures with thermal fluctuation or the mixed-phase of low- and high-density fragmentation. Its symmetry is the C_*v*2_ group; therefore, an oxygen atom tends to find four neighbors to form a stable tetrahedron, but the repulsion between electron pairs on oxygen prevents, in the liquid phase, its stability.

The corresponding packing order follows, in its entire phase diagram except for highest *T* and *P*, the Pauling’s rule [66]. The size of liquid water molecules is essentially stable despite fluctuations in both the O:H non-covalent bond lengths and bond angles. The average intermolecular distance changes only at the transition to the solid phase, when the system assumes an ordered structure and is therefore lower in density than the liquid.

Despite these thermal fluctuations, the Ice Rule extension results for both bulk liquid and ice in an ideal tetrahedron made by two equivalent molecules and between them four identical O:H-O bonds of different orientations. It is possible (see, e.g., ref. [67]) to have a direct relation between the water density and the distance $d_{OO}$ by considering the molecular mass *M* with $\rho =M/a^{3}=$ 1 (gcm^−3^) at 4 °C (under the atmospheric pressure). This defines unambiguously the density-dependent molecular separation, $d_{OO}$, and the next nearest-neighboring distance $\sqrt{2a}$ (in Å). Thus, we have $d_{OO}=\sqrt{3a}/2=2.6950\rho^{-1/3}$ and $\sqrt{2a}=4.4001\rho^{-1/3}$. By using these simple relations and the experimental density data, the intermolecular distance between two first neighbors water molecules can be evaluated.

**Figure 7:**
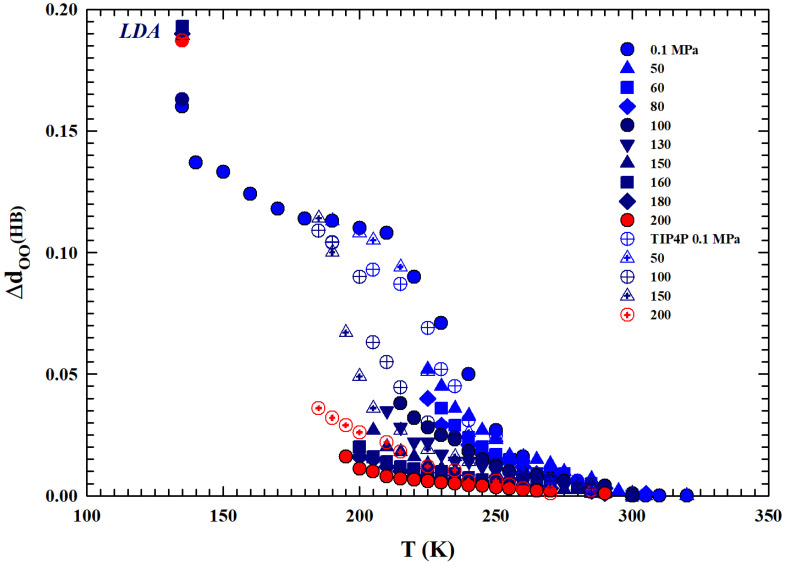
The differences, with respect to the corresponding $T^{*}$ values, of the mean oxygen–oxygen distances $d_{OO}$ ($\Delta d_{OO}$) between water molecules are reported. Specifically, we consider a wide temperature range from the thermally stable regions (330 K) to the low-density amorphous phase (135 K), including all the supercooled liquid regions. Values for different isobars from 0.1 to 200 MPa and the values obtained through the TIP4P MD simulation (open symbols) are shown. Although the latter represent the average distance between oxygen atoms for water molecules, they show similar thermal behavior. The behavior of the data indicates an evolution of the liquid, upon cooling, toward the LDA phase.

For the different isobars considered (from 0.1 to 200 MPa, together with the experimental data of the TIP4P MD [29,30] also reported), Figure 7 reports the difference of the molecular separations obtained at the different temperatures, $\Delta d_{OO}$, with respect to the corresponding values measured at $T^{*}$. Regarding the MD data reported here and calculated via the TIP4P model using a radial distribution function, it must be specified that they represent the average distance between oxygen atoms in water molecules. What emerges from the comparison between these data and those obtained here via the density values is a coincidence in their values, at all isobars considered, for all temperatures of the stable phase, while they show gradually increasing differences within the supercooled metastable phase. This is due to the known sensitivity of the model used in regions characterized by metastability; in such cases, it is preferable to use other models, such as E3B3 [31]. However, beyond this situation, both datasets show a similar thermal evolution. The data at 135 K correspond to the values of the low-density amorphous phase (LDA), values towards which the different isobars of the liquid phase evolve. In addition, as can be observed, these quantities increase as the temperature decreases with strong differences in their rates as the pressure increases. We can summarize this by saying that it is all due to the thermodynamic effects on the system polymorphism, created by the HB in particular, the increasing ratio under supercooling between the two phases LDL and HDL, and also the pressure effects on this.

## 3. Materials and Methods

Our study focused on the water system, in its liquid phase, in order to highlight the specificity of its particular thermodynamic singularity ($T^{*}$). It was arranged by considering essentially the isobars of a large lot of literature data: the density, the constant pressure specific heat and the transport functions such as self diffusion and rotational relaxation times. All this over a wide range of pressures and temperatures including both the stable and metastable (supercooled) phases. As mentioned, the data considered essentially concern bulk water, although the experimental accessibility to the metastability region was amplified using confined or emulsified water.

As underlined, the nuclear magnetic resonance, NMR, technique was used in order to obtain data on the transport functions ($D_{S}$ and $\tau_{\theta }$) as well as those on the specific heat. In this last case $C_{P}$ was also obtained through the Adam–Gibbs relation and compared with those measured through a differential calorimeter. The isobarsi of the Isothermal Compressibility and the Isobaric Thermal Expansivity were then obtained as the thermodynamic derivatives (of the isotherms and isobars) of the density. Finally, the Intermolecular Oxygen–Oxygen Distances, $d_{OO}$, where obtained through the extension of the Ice Rule to the liquid phase.

## 4. Conclusions

We considered the experimental water density isobars in a large area of the phase diagram including data from an MD simulation [29,30] and the measured data in the two characteristic glasses, LDA and HDA [5,6,7,8,9,42]. The temperature range considered was from 100 to 380 K, while the pressures considered ranged from atmospheric (0.1 MPa) to 800 MPa. Special attention was paid to the liquid phase in the supercooled region below the melting temperature $T_{m}$ and to a particular temperature $T^{*}≃315\pm 5$ K where the compressibility ($K_{T}\left(T,P\right)$) and expansivity ($\alpha_{P}\left(P,T\right)$) show unique thermodynamic properties [34]. For the supercooled region, we carefully considered the polymorphism of the system, characterized as is known by the presence of two liquid phases: LDL (due to HB) and HDL.

As in complex liquids, in water, the interaction process originates disordered correlated regions influencing the transport functions by means of a dynamic crossover. In water, this situation is entirely due to the specificities of the HB bond, which means tetrahedral networking for the system structure. These considerations indicate that a change in the water dynamics occurs at $T^{*}$, the temperature at which networking takes place. Change observable through a transition from a high-*T* region is characterized by an Arrhenius behavior; this change is to another typical glass-forming liquid system in which the temperature decrease gives rise to increasing intermolecular interactions and correlations with growing activation barriers resulting in super-Arrhenius behavior characterized by multirelaxations. Thus, this clustering process with increasingly extended structures (LDL) in the subcooled metastable phase is, unlike the disordered phase typical of high temperatures (HDL), strongly dependent on pressure.

Taking all this into account, we focused on this crossover temperature to verify how it determines the physical chemistry of water. We then carefully examined, over a large region of its phase diagram, the isobars of various thermodynamic functions such as the specific heat $C_{P}\left(P,T\right)$, the self-diffusion $D_{S}\left(P,T\right)$, and the rotational relaxation times $\tau_{\theta }\left(P,T\right)$, finding full suggestion on the universality of this specific temperature. Obviously, this is a suggestion and not a certainty, which could be supported by new studies, especially on the dynamic properties of the system in its metastable phases, both theoretical and experimental, also using the suggestions of molecular dynamics studies.

Finally, we paid attention to the distance $d_{OO}$ (the intermolecular distance between two first neighbors water molecules) evaluated from the density isobars, demonstrating that these intriguing realities are entirely due to the polymorphism of the liquid in its supercooled metastable phase. 

## Data Availability

The original contributions presented in this study are included in the article. Further inquiries can be directed to the corresponding author.
